# Crystal Digital PCR™ Enables Precise Quantification of Species Abundance in Microbial Mixtures

**DOI:** 10.3390/microorganisms13112592

**Published:** 2025-11-14

**Authors:** Louis Delecourt, Mila Reset, Lionel Bertaux, James Sturgis, Yann Denis, Marie-Thérèse Giudici-Orticoni, Magali Roger, Christophe Bordi

**Affiliations:** 1 LISM, IMM, Aix-Marseille Univ and CNRS, 13402 Marseille, France; loudel@dtu.dk (L.D.); mreset@imm.cnrs.fr (M.R.); sturgis@imm.cnrs.fr (J.S.); 2 BIP, IMM, Aix-Marseille Univ and CNRS, 13402 Marseille, France; giudici@imm.cnrs.fr; 3 LCB, IMM, Aix-Marseille Univ and CNRS, 13402 Marseille, France; lbertaux@imm.cnrs.fr; 4 Platform Transcriptome, IMM, Aix-Marseille Univ and CNRS, 13402 Marseille, France; ydenis@imm.cnrs.fr

**Keywords:** digital PCR, bacterial counting, quantification of mixed populations, consortium

## Abstract

Accurate bacterial quantification is critical in many biological fields, from clinical diagnostics to environmental microbiology. Here, we establish a robust workflow for absolute quantification of bacterial species within mixed communities using Crystal Digital PCR^TM^. Using a synthetic consortium of *Clostridium acetobutylicum* and *Nitratidesulfovibrio vulgaris*, we optimized primer design for species-specific detection and demonstrated that Crystal Digital PCR^TM^ enables reliable quantification of low-abundance species, down to a 1:10,000 ratio. We further show that the presence of one species does not interfere with the quantification of another. Finally, we demonstrate that Crystal Digital PCR^TM^ can also be used to determine plasmid-to-chromosome copy number ratios in bacteria carrying megaplasmids.

## 1. Introduction

Performing bacterial counting plays a crucial role in environmental management, industrial biotechnology, and public health by providing essential information about bacterial populations and their impact on different systems. In environmental science, bacterial quantification is fundamental for monitoring the quality of treated wastewater and drinking water, ensuring they remain free from hazardous bacterial contaminations. Moreover, tracking microbial communities involved in pollutant bioremediation is critical for assessing and optimizing their role in breaking down harmful substances, such as hydrocarbons, plastics, or heavy metals. Beyond environmental applications, bacterial communities are important for industrial biotechnology [1,2]. Multi-species microbial consortia can be engineered to harness beneficial interactions, leading to systems with emergent properties such as enhanced metabolic functionality, stability, or productivity. For example, synthetic microbial communities have been increasingly used for their ability to accumulate, metabolize, and degrade various compounds, including cellulose [3], plastics, and toxic heavy metals [4]. Additionally, they play a crucial role in the sustainable production of commodity chemicals [5] and pharmaceuticals [6], offering eco-friendly alternatives to traditional chemical synthesis. A remarkable example of microbial cooperation is biomass degradation in anoxic environments, where diverse microorganisms work together to break down complex organic matter and generate biogas. Inspired by this natural process, a simplified bacterial consortium was developed, consisting of *Clostridium acetobutylicum* and *Nitratidesulfovibrio vulgaris*. This engineered microbial system fosters synergistic interactions that enhance biohydrogen production, highlighting the potential of microbial communities in advancing sustainable energy solutions [7,8,9].

Thus, bacterial communities constitute a valuable resource in biotechnology, with tremendous potential for driving sustainable innovation. Their exceptional metabolic versatility and adaptability enable a wide range of applications, from vaccine development and environmental cleanup to bioplastic production and biofuel synthesis. However, although the significance of multispecies communities in various applications is well-established, understanding these communities remains challenging. The lack of detailed knowledge about microbial communities, particularly regarding their composition, stability, and evolution, remains a major obstacle to fully understanding their functions, thereby hindering rational engineering and better control of synthetic ecosystems. Indeed, microbial diversity, which defines the genetic potential within an ecosystem, is a key factor influencing its function, stability, and evolution. In addition to genetic diversity, bacterial evenness—the relative abundance of each species—an important aspect of ecological diversity, also plays a crucial role in shaping ecosystem dynamics [10,11]. It is therefore crucial to accurately quantify the different species within an ecosystem—whether natural, clinical, or industrial—in order to monitor changes in population sizes over time and/or in response to environmental fluctuations. This provides valuable insights into the dynamics of bacterial communities and their interactions, helping to improve our understanding of ecosystem function, optimize biotechnological processes, and ensure safety and quality across various sectors. The main methods of bacterial quantification include culture on solid media, quantitative PCR (qPCR), flow cytometry, and metagenomics, each with distinct advantages and limitations [12,13,14,15]. Culture on solid media is one of the oldest methods, involving the growth of bacteria on nutrient-rich surfaces and counting the resulting colonies [16]. This method is simple, cost-effective, permits identification of living bacteria in samples and allows visual differentiation of bacteria. However, it is a time-consuming approach since many bacteria can exhibit long generation times and is, of course, limited to cultivable bacteria [17,18] and complex to implement in anaerobic or extremophilic growth conditions [19]. A second method very frequently used for counting bacteria in a sample is qPCR that measures the number of copies of specific bacterial genes, offering precise, rapid quantification and high sensitivity [20,21]. However, in qPCR, the quantification relies on a standard curve generated from known concentrations of a DNA. This curve is essential for converting the fluorescence signal into quantitative data, but it requires meticulous preparation and validation [22]. Variations in PCR efficiency and amplification conditions can affect the accuracy of the standard curve, leading to potential inaccuracies in quantification [23]. Moreover, qPCR is susceptible to inhibition from contaminants or complex sample matrices, which can further impact the reliability of results [20]. These factors restrict the sensitivity and precision of qPCR, particularly when quantifying low-abundance targets or when dealing with challenging sample types. Additionally, qPCR cannot differentiate between live and dead bacteria, as it quantifies DNA regardless of cell viability [24]. Flow cytometry is another powerful method for quantifying bacterial populations, offering rapid analysis and multiparametric capabilities to measure various physical and biological characteristics of cells simultaneously and can distinguish live from dead cells using specific dyes [25]. Flow cytometry’s high sensitivity allows detection of rare events, making it effective for quantifying bacteria even at low concentrations [26]. However, this counting method is facing challenges when distinguishing between closely related bacteria solely based on cell shape and granularity [27]. To overcome this limitation, specific fluorescent labeling is crucial. This method involves tagging bacterial cells with fluorescent markers that bind to particular cell surface antigens or components, enabling differentiation based on unique fluorescence patterns [28,29]. However, the effectiveness of these markers can vary depending on the bacterial species and the conditions of the sample (anaerobic conditions for example) [25]. Another drawback of fluorescence-based cell counting is that developing species-specific antibodies is costly and time-consuming, which can limit its practical application. Despite these limitations, flow cytometry remains widely used to analyze mixed microbial communities, which is a primary goal in many studies. Nevertheless, a key limitation of the technique is that distinguishing closely related species or accurately identifying multiple species in complex mixtures can be challenging, as overlapping fluorescent signals may complicate precise species discrimination [30]. Finally, Metagenomics is a powerful approach for studying bacterial diversity and composition in complex environmental samples and offers comprehensive insights into bacterial diversity and potential metabolic capabilities [31]. However, it is costly, generating large datasets requiring advanced bioinformatics and cannot distinguish between viable and non-viable bacteria [32].

Over the past two decades, various PCR-based techniques have been developed, including a new approach known as Digital PCR (dPCR) [33,34,35]. dPCR is a DNA quantification method that enables extremely precise and sensitive detection and quantification of nucleic acid molecules. This makes it particularly useful for detecting low-abundance targets. Unlike quantitative PCR (qPCR), which measures the accumulation of an amplified product in real time, digital PCR partitions a DNA sample into thousands of small individual reactions, some containing target DNA molecules and others not. Each partition acts as an independent PCR reaction. This partitioning process ensures that some reactions contain the target DNA molecules, while others do not. After amplification, the positive partitions, which emit a fluorescent signal, are counted to determine the absolute quantity of the target DNA initially present. One of the key advantages of dPCR is its precision and sensitivity; it provides absolute quantification without the need for standard curves, which is a requirement in traditional qPCR. Additionally, dPCR is less affected by PCR inhibitors, which are common in complex samples, leading to higher accuracy and reliability. Digital PCR (dPCR) could be divided into main methods: Digital PCR using droplets and Well-based digital PCR. Digital using droplet (ddPCR) divides the sample into thousands of nanoliter-sized droplets which can be separated and stabilized in an oil-based emulsion. After thermal cycling, the droplets pass through a capillary or detection system where they are individually counted and analyzed to determine whether they are positive (containing the target DNA) or negative (no target DNA). Well-based digital PCR, on the other hand, partitions the sample into hundreds or thousands of individual wells (e.g., on a chip or plate), with each well-functioning as a separate PCR microreaction. Fluorescence is then measured to determine which wells show successful amplification of the target sequence. Regardless of whether droplet-based or well-based digital PCR is used, the fundamental process is the same: the sample is partitioned into individual reaction units, and fluorescence is evaluated to identify positive reactions, highlighting the methodological similarity between the two approaches.

In this study, we aim to establish best practices for employing Crystal Digital PCR^TM^ to provide a quantitative and absolute assessment of the number of individuals within a bacterial community, using the consortium formed by *N. vulgaris* and *C. acetobutylicum* as models. This work includes establishing quantification limits for a species within the population, investigating the impact of one species on the quantification of another, and finally utilizing this technique to measure the number of plasmids and chromosomes found in the cytoplasm of a bacterial species.

## 2. Materials and Methods

### 2.1. Strains, Media and Growth Conditions

*N. vulgaris* Hildenborough and *C. acetobutylicum* ATCC824 strains used in this study were sourced from NCIMB biotechnology (NCIMB 8303) and Henry-Pierre Fierrobe’s lab (LCB, UMR7283 CNRS-AMU, Marseille, France), respectively. Strains were grown to steady state in Hungate tubes under anaerobic conditions at 37 °C, in Starkey medium (SKY) containing 40 mM lactate and 28 mM sulfate for *N. vulgaris* [36] and 2YTG medium for *C. acetobutylicum* [7]. The growth medium glucose–yeast extract (GY) used for the consortium was prepared with 14 mM glucose and 0.1% yeast extract, supplemented with the same inorganic nutrients as in SKY medium [7]. GY medium was inoculated with either washed *N. vulgaris*, *C. acetobutylicum*, or both strains to reach different ratios based on their absorbance at 600 nm. All experiments were performed at least in triplicate.

### 2.2. DNA Extraction from Bacterial Culture

DNA from *N. vulgaris* and *C. acetobutylicum* pure cultures were extracted using the Wizard^®^ Genomic DNA Purification Kit (Promega A1120, Promega Corporation, Madison, WI, USA). Briefly, 10 mL of each culture was centrifuged at 10,000× *g* for 10 min. The resulting pellet was resuspended in 600 µL of 50 mM EDTA (ethylenediaminetetraacetic acid) with 10 mg·mL^−1^ lysozyme and incubated at 37 °C for 1 h. DNA extraction was then carried out according to the manufacturer’s instructions. The final elution step was performed with 100 µL of pre-heated (55 °C) nuclease-free water. DNA extraction from the Controlled Mixed Community was performed using the GenElute^TM^ Bacterial Genomic DNA Kit (Sigma-Aldrich, NA2110, Saint Louis, MO, USA) following the manufacturer’s recommendations. Briefly, 1 mL of the mixed community was centrifuged at 10,000× *g* for 15 min directly after inoculation. The supernatants were removed, and the pellets were stored at −20 °C until use. To improve the lysis step, the Gram-Positive Bacterial Preparation protocol was selected and optimized as follows: (1) 200 µL of lysozyme solution (human lysozyme L1667, Sigma, Saint Louis, MO, USA) at a concentration of 10 mg·mL^−1^ was used; (2) the incubation with lysis solution C was extended to 20 min during the “Lyse Cells” step; and (3) elution was performed using 50 µL of pre-heated nuclease-free water (55 °C). DNA concentrations (in ng·µL^−1^) and quality were determined, using a Nanodrop^TM^ 2000c spectrophotometer (Thermo Scientific, Waltham, MA, USA).

### 2.3. Identification of Species-Specific Genes and Primer Design

Species-specific genes for *C. acetobutylicum* and *N. vulgaris* were identified using OrthoFinder (version 3.1; https://github.com/OrthoFinder/OrthoFinder installed locally, accessed on 26 October 2025). Protein sequences were extracted from GenBank files AE001437.1 (*C. acetobutylicum*) and AE017285.1 (*N. vulgaris*) using Biopython and converted to FASTA format. OrthoFinder grouped proteins into orthologous clusters, and genes unique to each genome were identified from clusters containing proteins from only a single genome. DNA sequences of these unique genes were then used to design specific primers with Primer-BLAST (version 2.5.0; https://www.ncbi.nlm.nih.gov/tools/primer-blast/, accessed on 26 October 2025), selecting oligonucleotides with similar melting temperatures to ensure consistent amplification under identical PCR conditions.

### 2.4. PCR Experiment

Conventional PCR assays were carried out using a T100^TM^ thermocycler (BioRad, Hercules, CA, USA) with 75 ng·µL^−1^ of bacterial DNA, 10 units of GO-Taq^®^ polymerase, 1 mM dNTPs (New England Biolabs, Ipswich, MA, USA), and 0.5 µM of the primers specified in Table 1. The PCR reaction was conducted with the following cycling parameters: 95 °C for 2 min, followed by 40 cycles of 95 °C for 30 s, 58 °C for 30 s, and 72 °C for 30 s, followed by a final extension at 72 °C for 3 min. PCR products were separated on an agarose gel, stained with GelRed^®^ (Hayward, CA, USA), and visualized under UV light. Quantitative PCR (qPCR) assays were performed on CFX96 Touch^TM^ Real-Time PCR Detection System using SsoAdvanced^TM^ Universal SYBR Green^®^ Supermix (Bio Rad, Hercules, CA, USA) and 0.5 µM of the primers. The cycling parameters for the qPCR were as follows: 98 °C for 2 min, followed by 45 cycles of 96 °C for 20 s and 60 °C for 10 s. This was concluded with a final step at 95 °C for 3 min prior to establishing the melting curves. Crystal Digital PCR^TM^ reactions were conducted in the Geode^TM^ thermocycler (Stilla Technologies, Villejuif, France), associated with the Fluigent^®^ FLPG pressure generator (Fluigent, Le Kremlin-Bicêtre, Franc). In a final volume of 25 µL, the DNA samples were mixed with 9.375 µL of 2× PerfeCTa^®^ qPCR ToughMix^®^ (Quantabio, Beverly, MA, USA) solution, supplemented with 1 µL of Alexa 647 (Thermo Scientific, Waltham, MA, USA) at a concentration of 0.02 mg·mL^−1^, 1.9 µL of 20X EvaGreen^®^ DNA-binding dye (Biotium, Fremont, CA, USA), and 0.5 µL of primers at a concentration of 20 µM. Once the reagents were added to the samples, a 25 µL deposit was made in the chamber entries of the Sapphire Chips^TM^ (Stilla Technologies, Villejuif, France). The amplification steps include partitioning the samples at 40 °C, followed by denaturation at 95 °C for 3 min. This is followed by 45 cycles of denaturation at 95 °C for 10 s, then a hybridization/elongation step at 60 °C for 15 s. After amplification, three cycles of pressure at 50 mbar to better separate the droplets were made and the Sapphire Chips^TM^ were carefully transferred to the Prism3 chip^TM^ reader (Stilla Technologies, Villejuif, France) for analysis. The Crystal Reader^TM^ and Crystal Miner^TM^ software (version 4.0.10.3) were used for reading and analyzing the chips.

## 3. Results

### 3.1. Primer Selection and Specificity Testing for Crystal Digital PCR^TM^

Crystal Digital PCR^TM^ belongs to endpoint PCR techniques, where the final product is detected by a fluorescent intercalating molecule, such as EVAGreen, embedded in the double-stranded DNA obtained at the end of the PCR reaction. Nowadays, high-sensitivity fluorescence cameras permit the detection of weak fluorescence emissions, and methods like Crystal Digital PCR^TM^ require very well-designed primers to avoid any nonspecific PCR products or primer dimer formation that could combine with the fluorescence dye and create false-positive results. Moreover, in the objective of using this technique to quantify bacterial species in a multispecies population, it is also crucial to avoid any cross-hybridization of primers between bacterial genomes. To demonstrate the utilization of Crystal Digital PCR^TM^ for absolute quantification of a mixed bacterial population, we used a synthetic consortium composed of Gram-negative *N. vulgaris* and the Gram-positive *C. acetobutylicum* as a study model. In this consortium, the two bacteria can interact and exchange metabolites, allowing them to survive under nutritional stress [9].

Firstly, to limit primer cross-hybridization between bacterial genomes, we began by identifying specific genes of each bacterium using the OrthoFinder v3.1 [37], which allows the identification of common, orthologous, paralogous and unique genes between the genomes of *N. vulgaris* and *C. acetobutylicum (*see Section 2). Through this approach, we identified *DVU_0169* (ABC transporter substrate-binding protein) and *CA_C0825* (glycoside hydrolase family 5 protein) genes as specific to *N. vulgaris* and *C. acetobutylicum* genomes, respectively. These genes lack paralogs within their own genomes and do not have orthologs in other genomes. The DNA sequences of these genes were then used to design specific primers using the Primer-BLAST website, with careful attention to selecting oligonucleotides with similar melting temperatures to ensure consistent amplification under identical reaction conditions during Crystal Digital PCR^TM^. Even though PCR efficiency and amplicon product size are less restrictive as for Crystal Digital PCR^TM^ than they are for qPCR, we chose primers yielding amplicon sizes around 200–250 bp and showing no homodimerization (Table 1).

**Figure 1:**
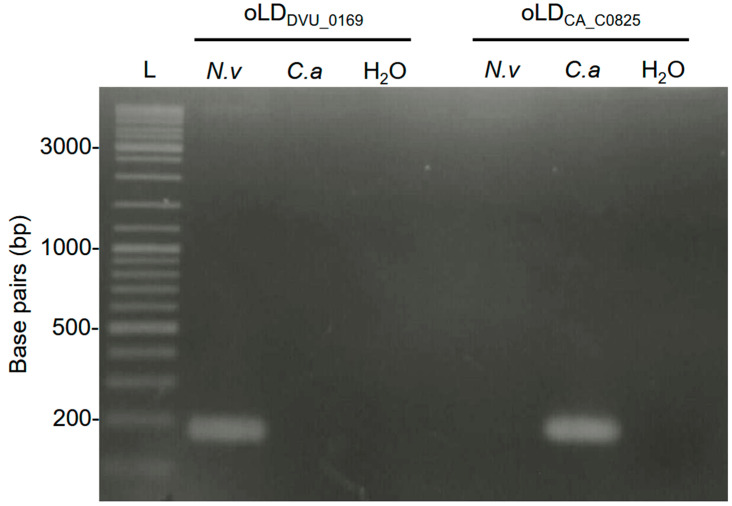
Agarose gel electrophoresis (2% agarose) of PCR-amplified products using species-specific primer sets. Results obtained with the oLDDVU_0169 primer pair on *N. vulgaris* (*N.v*), *C. acetobutylicum* (*C.a*), and water (H_2_O) are shown, together with those from the oLDCA_C08025 primer pair under identical conditions. Lane L contains 5 µL of Thermo Scientific™ GeneRuler™ DNA Ladder Mix.

Once the primers were designed, we began to verify their specificity to detect only their target genomes, as well as the absence of nonspecific PCR products including primer dimers. To validate our primer pairs, the genomic DNA of *N. vulgaris* and *C. acetobutylicum* were purified and used as templates for PCR, qPCR, and Crystal Digital PCR^TM^ approaches. In the PCR, a concentration of 75 ng·µL^−1^ of genomic DNA from *N. vulgaris* and *C. acetobutylicum* was used as the template for amplification with the designed primers oLD_DVU_0169_ and oLD_CA_C0825_ (see Section 2). Subsequently, the PCR products were analyzed by agarose gel electrophoresis and visualized under UV light using GelRed dye. As shown in Figure 1, the primers for *N. vulgaris* and *C. acetobutylicum* successfully amplified only their respective target genomes, with no cross-amplification observed on the other genome. Furthermore, no amplification was observed in the water control, suggesting that none of the primer pairs formed dimers during the reaction.

**Figure 2:**
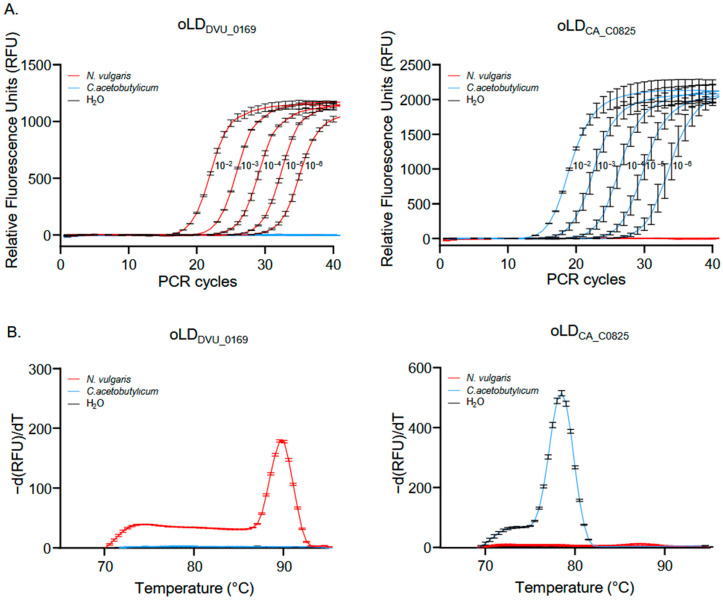
Verification of primers pair specificity using qPCR. (**A**) Amplification curves generated by oLD_DVU_0169_ (**left panel**) and oLD_CA_C0825_ (**right panel**) on *N. vulgaris* and *C. acetobutylicum* genomes at different concentrations, with water (H_2_O) as a negative control. (**B**) Melting curves of the PCR products obtained at the end of qPCR with primers on *N. vulgaris*, *C. acetobutylicum*, and water (H_2_O) with oLD_DVU_0169_ (**left panel**) and oLD_CA_C0825_ (**right panel**). Mean values and standard deviations (error bars) are shown, calculated from three independent biological replicates.

To validate these results and to assess whether DNA concentration affects the specificity and selectivity of our primers, a qPCR was conducted. An initial concentration of 500 ng·µL^−1^ of *N. vulgaris* and *C. acetobutylicum* DNA genomes was serially diluted from 10^−2^ to 10^−6^, followed by qPCR using our primers (see Section 2). As shown in Figure 2A, the *N. vulgaris* and *C. acetobutylicum* primers produce PCR products only in the presence of their target genomes, regardless of the initial DNA concentration, while no PCR products were detected in the water control. Furthermore, the analysis of the melting curves (Figure 2B) for the final PCR products reveals that only one PCR product is detected in the presence of the target genomes corresponding to our primers.

**Figure 3:**
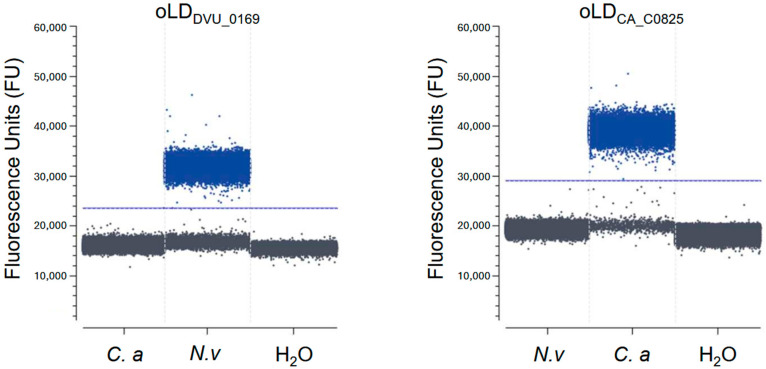
Specificity assay of primer pairs using Crystal Digital PCR^TM^. Number and fluorescence intensities of droplets at the end of the Crystal Digital PCR^TM^ reaction generated by oLD_DVU_0169_ (**left panel**) and oLD_CA_C0825_ (**right panel**) on *N. vulgaris* (*N.v*) and *C. acetobutylicum* (*C.a*) genomes at different concentrations, with water (H_2_O) as a negative control.

Moreover, no significant PCR products or primer dimerization were observed in the water control or in the presence of non-target genomic DNA. Finally, to evaluate the specificity of our primers in detecting only their target genomes, a Crystal Digital PCR^TM^ was conducted. Our primer pairs were mixed with either water or bacterial genomes, and positive droplets were fluorescently detected and counted (Figure 3). The presence of positive droplets only when the primers are in the presence of their target genome confirms that the designed primers can specifically detect their target genome (*N. vulgaris* or *C. acetobutylicum*) without generating any non-specific amplicons or primer dimers detectable by digital PCR on the other genome.

### 3.2. Assessment of Crystal Digital PCR^TM^ Sensitivity for Quantifying Mixed Genomes in Samples

**Figure 4:**
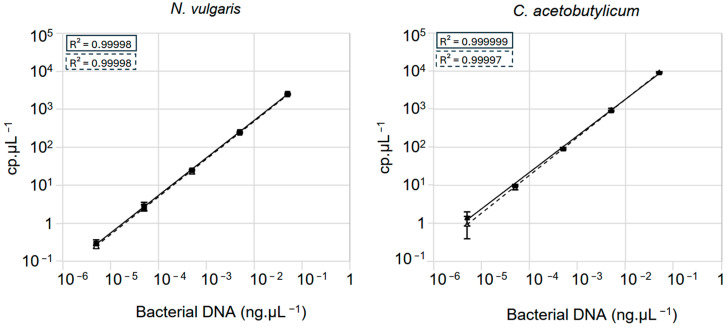
Detection threshold test in the presence or absence of exogenous DNA. The linearity of Crystal Digital PCR^TM^ quantification was evaluated by analyzing serial dilutions of DNA extracted from *N. vulgaris* (**left panel**) and *C. acetobutylicum* (**right panel**). Results are presented for both the presence (dashed line/box) and absence (solid line/box) of exogenous DNA at a fixed concentration of 0.05 ng/µL. Mean values and standard deviations (error bars) are shown, calculated from three independent biological replicates.

In mixed bacterial populations, the relative abundance of each species may vary, and some of them may be in the minority, present in only a few individuals among the others. Digital PCR technologies such as Crystal Digital PCR^TM^ are described as being able to quantify genome copy numbers as low as 2 cp·µL^−1^ [38]. We therefore assessed the detection limit of Crystal Digital PCR^TM^ for quantifying pure or mixed DNA samples, using our primer pairs on *N. vulgaris* and *C. acetobutylicum* DNA. We began by serially diluting, in tenfold steps, an initial quantity of 0.05 ng·µL^−1^ of *N. vulgaris* and *C. acetobutylicum* DNA until reaching a final concentration of 5 fg·µL^−1^. Then, these DNA samples were quantified by Crystal Digital PCR^TM^ using the appropriate primers. The results demonstrate that for both primer pairs, we can detect a number of genome copies in the samples as low as 0.29 cp·µL^−1^ for *N. vulgaris* and 1.43 cp·µL^−1^ for *C. acetobutylicum* (Figure 4 solid line). Additionally, we also observe excellent linearity in DNA quantity in the samples, with a regression line showing an R^2^ value close to 1 (Figure 4). To determine whether the presence of another DNA genome in the reaction could alter the number of genome quantifications in the samples, we started to dilute the *N. vulgaris* genome to an amount ranging from 0.05 ng·µL^−1^ to 5 fg·µL^−1^ in a solution containing a fixed amount of *C. acetobutylicum* genome at 0.05 ng·µL^−1^. We then quantified the *N. vulgaris* DNA in these samples using oLD_DVU_0169_ by Crystal Digital PCR^TM^. As shown in Figure 4 (left panel, dashed line) the number of copies of the *N. vulgaris* genome in the presence of fixed amount *C. acetobutylicum* DNA is identical to the results obtained with *N. vulgaris* alone in the reaction, regardless of the ratio between the amount of *N. vulgaris* and *C. acetobutylicum* DNA in the reaction. A similar result was obtained when the *C. acetobutylicum* genome was diluted from 0.05 ng·µL^−1^ to 5 fg·µL^−1^ in a solution containing 0.05 ng·µL^−1^ of *N. vulgaris* DNA (Figure 4; right panel, dashed line).

Overall, our results demonstrate that Crystal Digital PCR^TM^ technology can detect low quantities of DNA, down to 2 cp·µL^−1^, in mixed bacterial population samples. Furthermore, the presence of another DNA species in the reaction does not impact the quantification of the target DNA, highlighting the effectiveness of Crystal Digital PCR^TM^ for accurately quantifying genomes in complex samples.

### 3.3. Bacterial Quantification in a Controlled Mixed Community

**Figure 5:**
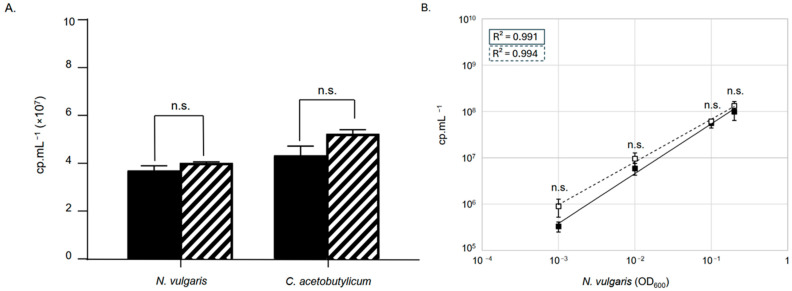
Bacterial quantification in pure or mixed culture. (**A**) Quantification of 0.1 OD_600_ units of *N. vulgaris* and *C. acetobutylicum* in pure cultures (black bar) or in mixed cultures at a 1:1 ratio (hashed bars). (**B**) Quantification of varying amounts of *N. vulgaris*, ranging from 0.2 to 0.001 OD_600_ units, in pure culture (solid line/box) or mixed with a fixed amount of 1 OD_600_ unit of *C. acetobutylicum* (dashed line/box). Mean values and standard deviations (error bars) are shown, calculated from three independent biological replicates. Statistical significance was assessed using the Wilcoxon–Mann–Whitney test; n.s. referred as not-significant.

To determine whether the Crystal Digital PCR^TM^ approach allows the quantification of different bacterial species within a bacterial consortium, we used the synthetic model consortium made of *N. vulgaris* and *C. acetobutylicum*. The aim is to assess whether we could quantify each bacterial species within the consortium and whether the presence of one organism influenced the detection of the other. For this, we created consortia with controlled bacterial proportions and tested their quantification using Crystal Digital PCR^TM^. Thus, the bacteria *N. vulgaris* and *C. acetobutylicum* were grown in SKY and 2YTG media, respectively, for 10 h until they reached the stationary growth phase (see Section 2). The optical density at 600 nm of each culture was measured, and then, they were diluted to an OD_600_ of 0.1 in a final volume of 10 mL GY medium. Three controlled consortia of *N. vulgaris* and *C. acetobutylicum* were prepared, each with a final volume of 10 mL and containing 0.1 units of optical density at 600 nm of each organism. Additionally, three tubes of 10 mL of pure cultures of *N. vulgaris* and *C. acetobutylicum* at 0.1 OD_600_ were prepared as controls. Then, the total DNA from 1 mL of pure and mixed cultures was extracted using the GenElute Bacterial Genomic DNA kit, following the manufacturer’s instructions. Then, the primer pairs oLD_DVU_0169_ and oLD_CA_C0825_ were used to quantify the genome copy number of *N. vulgaris* and *C. acetobutylicum*, both in pure cultures and in 1:1 co-culture. As shown in Figure 5A, mean genome quantities of 3.7 × 10^7^ cp·mL^−1^ and 4.3 × 10^7^ cp·mL^−1^ were detected in pure cultures at an OD_600_ of 0.1 for *N. vulgaris* and *C. acetobutylicum*, respectively. Similar quantification performed on the co-culture yielded mean values of 4.0 × 10^7^ cp·mL^−1^ and 5.2 × 10^7^ cp·mL^−1^ for *N. vulgaris* and *C. acetobutylicum*, respectively (Figure 5A). Thus, there is no significant difference in the number of genomes detected for each of the two organisms, whether in pure culture or co-culture, suggesting that the presence of one does not influence the quantification of the other. Next, we determined the detection limit for bacteria in culture and whether this detection is influenced by the presence of the other member of the consortium. We prepared several co-cultures, each with a final volume of 10 mL. These co-cultures contained a fixed OD_600_ of 1 for *C. acetobutylicum* along with a serial dilution of *N. vulgaris*, with OD_600_ values ranging from 0.2 to 0.001. For comparison, we also prepared control reaction tubes of 10 mL containing only *N. vulgaris*, serially diluted from 0.2 to 0.001. Subsequently, we extracted the total DNA from 1 mL of both the pure and mixed cultures and evaluated the number of *N. vulgaris* genomes using the primers oLD_DVU_0169_ (Figure 5B). The results obtained show a linear quantification with a regression coefficient R^2^ close to 1 for the number of *N. vulgaris* genomes present in the cultures, whether they contain a fixed amount of *C. acetobutylicum* or not. We do not observe a significant difference in the quantification of *N. vulgaris* genomes between the presence and absence of *C. acetobutylicum* in proportions of 1:1 and 1:1000.

### 3.4. Plasmid Quantification in N. vulgaris and C. acetobutylicum Cultures

**Figure 6:**
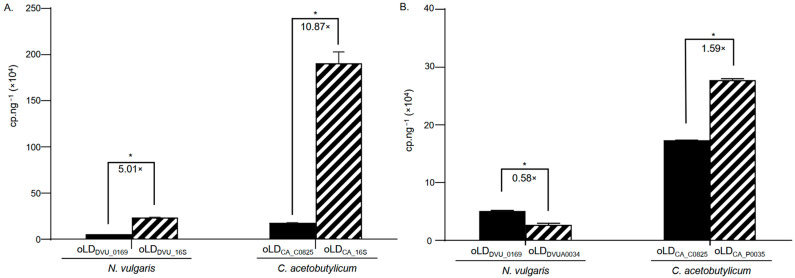
Quantitative comparison of the copy numbers of two distinct DNA sequences within the same DNA sample from *N. vulgaris* and *C. acetobutylicum*. (**A**) Quantification of unique genes (black bar) and multicopy 16S encoding genes (hashed bar) present in the same bacterial genome. (**B**) Quantification of chromosome copy numbers (black bar) and plasmid copy numbers (hashed bar). Mean values and standard deviations (error bars) are shown, calculated from three independent biological replicates. Statistical significance was assessed using the Wilcoxon–Mann–Whitney test, * referred *p* < 0.05.

Genome sequencing of *N. vulgaris* and *C. acetobutylicum* revealed the presence of a megaplasmid in each strain, with a respective size of 202,301 bp and 192,000 bp, respectively [39,40]. However, the ratio between the number of plasmids and the number of chromosomes in the cytoplasm of these bacteria remains unknown. Indeed, qPCR quantification approaches are limited for reliably comparing two different DNA sequences within the same sample due to the necessity that the targeted sequences are amplified with comparable efficiency. Additionally, this method requires calibration curves to estimate copy numbers, which may introduce biases, making it difficult to directly compare the copy number of one gene to another. Crystal Digital PCR^TM^, which offers absolute quantification independent of calibration curves and PCR efficiency, appears to be a highly suitable tool for addressing this type of question. To verify this, we used Crystal Digital PCR^TM^ to quantify the relative copy number of plasmids in *N. vulgaris* and *C. acetobutylicum* compared to the copy number of their respective genomes. We first validated the ability of Crystal Digital PCR^TM^ to detect differences in copy number between a single-copy gene and genes present in multiple copies on the same chromosome. Thus, we measured and compared the copy numbers of the *DVU_0169* and *CA_C0825* single copy genes of *N. vulgaris* and *C. acetobutylicum* relative to the multicopy numbers of their respective 16S rRNA encoding genes. A pair of PCR primers targeting the 16S rRNA of *N. vulgaris* (oLD_DVU_16S_) and *C. acetobutylicum* (oLD_CA_16S_) was designed and verified for specificity, following the different PCR approaches conducted for the primer pairs targeting *DVU_0169* and *CA_C0825* genes (Table 1). Then, an amount of 0.005 ng·µL^−1^ of DNA extracted from the strains of *N. vulgaris* and *C. acetobutylicum* was used, respectively, with primers oLD_DVU_0169_ and oLD_DVU_16S_, and oLD_CA_C0825_ and oLD_CA_16S_, to compare the copy number of the single-copy genes *DVU_0169* and *CA_C0825* to the copy number of the multicopy genes *DVU_16S* and *CA_16S*. As shown in Figure 6A, there is an average ratio of 5.02 and 10.87, respectively, for *N. vulgaris* and *C. acetobutylicum* between the copy number of unique genes and the copy number of multicopy genes. These results correspond to the expected ratios, since *N. vulgaris* and *C. acetobutylicum* have 5 and 11 copies of the gene encoding for 16S rRNA in their respective genomes. Since Crystal Digital PCR^TM^ allows for determining the quantitative ratio between different DNA sequences in the same sample, we used it to assess the ratio between the number of plasmids, and the number of chromosomes present in the cytoplasm of *N. vulgaris* and *C. acetobutylicum*. By following the same approach that allowed us to design the primer pairs oLD_DVU_0169_ and oLD_CA_C0825_, we designed the primers oLD_DVUA0034_ and oLD_CA_P0035_, targeting the genes *DVUA0034* and *CA_P0035* present on the megaplasmids of *N. vulgaris* and *C. acetobutylicum*, respectively (Table 1). These primers were then used in Crystal Digital PCR^TM^ on an extract of 0.05 ng·µL^−1^ of DNA from each bacterium to determine the quantitative ratio between the copy number of chromosomes and the copy number of plasmids. As shown in Figure 6B, there is an average ratio of 0.58 between the number of plasmids and the number of chromosomes in the cytoplasm of *N. vulgaris* and an average ratio of 1.59 for *C. acetobutylicum*, respectively.

## 4. Discussion

Bacterial consortia are involved in the major processes of life. In consequence a reliable, fast and efficient counting method is essential to understand and decipher the behavior of these consortia and to use them in biotechnological developments [41]. However, while it might seem like a straightforward task, it can prove to be quite challenging in certain contexts. This is particularly true when working with non-culturable bacteria, extremophiles, mixed cultures, or those forming aggregates or biofilms, which are often difficult to analyze using conventional techniques [19,42]. The results of this study demonstrate that the Crystal Digital PCR^TM^ method is an effective tool for detecting and quantifying bacterial populations within mixed communities. In this work, we demonstrated that it is possible to quantify down to a single bacterial genome per µL of sample. We also showed that this quantification is not affected by the presence of DNA from another organism, even when the ratio between the DNA quantities of the organisms is as low as 1:10,000 with using only biological triplicate. However, like all PCR-based techniques, this method requires attention to two key aspects to ensure robust results. The first point is the reproducibility of DNA extraction across different bacterial strains to ensure accurate and comparable quantification. This requires efficient and consistent bacterial lysis, as well as careful attention to avoid overloading silica membrane columns, which can become saturated and lead to underestimation of DNA yield. Maintaining extractions within a validated input range is key to preserving linearity between the number of cells and the amount of recovered DNA. Furthermore, when aiming to correlate genome copy number with actual cell counts, it is important to ensure that the quantified DNA truly reflects cellular DNA and not extracellular contamination. In this context, the removal of free DNA prior to extraction, for instance by DNase I treatment, can help improve the specificity of the measurement [24]. The second point of vigilance is that this method requires an extremely rigorous design of oligonucleotides following a highly stringent pipeline. Indeed, it is crucial that the oligonucleotides specifically hybridize to their target organism and do not form primer dimers. Here, we ensured specificity by selecting unique genes for each organism based on genomes comparison. However, this approach can also be applied to DNA sequences that are specific to a species and absent from other organisms, without targeting a particular gene. It can thus be used for intergenic regions or genetically added markers in a chromosome, allowing differentiation between different strains. This strategy can be particularly useful in Crystal Digital PCR^TM^ experiments to assess fitness differences between two variants of the same species, for example. The importance of this design increases with the complexity of the environment, particularly when many different organisms are present. Indeed, Crystal Digital PCR^TM^, as a final-point PCR technique, only provides information on the presence or absence of DNA amplification in a droplet. It does not allow for determining the size of the product, or the melting temperature of the amplification product within the droplet. Consequently, even minimal nonspecific amplification, caused by the formation of primer dimers or nonspecific primer hybridization to different genomes in the sample, can result in fluorescent droplets that skew the quantification results. However, this technique offers significant advantages over qPCR. Firstly, Crystal Digital PCR^TM^ demonstrates greater resistance to inhibitors, such as proteins or chemical compounds present in the sample, making it a reliable method for analyzing complex matrices [43,44,45]. Additionally, its capacity for absolute quantification enables direct comparison of different experiments without the need for normalization using standard curves, as required in qPCR. This feature also simplifies data comparison between laboratories or across extended time periods, as it eliminates the need for standardization using specific calibration curves. At last, dPCR offers higher sensitivity, enabling highly precise quantification at very low concentrations from a limited number of measurements in small samples, whereas qPCR, although theoretically capable of absolute quantification, requires a substantially larger number of data points to achieve comparable precision. In this study, we used the bacterial consortium composed of *N. vulgaris* and *C. acetobutylicum* as models. However, the quantification of organisms by Crystal Digital PCR^TM^ on populations can be adapted to various contexts, including, for example, bacterial competition experiments to evaluate interactions between different species, as well as fitness studies to determine if a strain that has acquired or lost a fragment of DNA or single nucleotide polymorphisms (SNPs) exhibits a fitness difference when co-cultured with the wild-type strain from which it is derived [46].

This work also demonstrates that Crystal Digital PCR^TM^ is an effective technique for quantifying the number of gene copies within the same genome, as illustrated by our results on the quantification of the 16S gene in the *C. acetobutylicum* and *N. vulgaris* strains. Thanks to Crystal Digital PCR^TM^, we were able to confirm, in accordance with predictions based on the genome annotations of these two organisms, the existence of a 5:1 ratio for *N. vulgaris* and an 11:1 ratio for *C. acetobutylicum* between the number of copies of the 16S genes and the single-copy genes *DVU_0169* and *CA_C0825*. This allowed us to determine for the first time the plasmid copy number in *C. acetobutylicum* and *N. vulgaris* relative to the chromosome copy number. The results show that the proportion between the number of plasmids and chromosomes is not equivalent in the cytoplasm and may, in some cases, reflect variability in the ratio of plasmid copies to chromosome copies within individuals of the same population. For *C. acetobutylicum*, an average plasmid-to-chromosome ratio of 1.5 was observed. This imbalance may reflect heterogeneity in plasmid copy number among cells within the population for instance, some cells may maintain a 1:1 ratio of plasmid to chromosome, while others contain additional plasmid copies. Another plausible explanation involves differences in replication timing between the chromosome and the plasmid. Plasmid replication is known to occur independently and asynchronously with the bacterial cell cycle [47,48], meaning that plasmid duplication is not strictly coordinated with chromosomal replication. As a result, cells at different stages of growth may transiently contain more or fewer plasmid copies relative to their chromosomal content. When measured across an entire population, this asynchrony can lead to apparent deviations from a 1:1 ratio even if each cell maintains a stable average number of plasmids. Such variability is consistent with population-level measurements, which integrate signals from cells in diverse replication states. For *N. vulgaris*, the observed ratio of 0.5 could arise from several non-exclusive explanations. One possibility is that cells harbor multiple chromosomal copies per cell, as previously estimated for *N. vulgaris* (~4 genomes per cell) [49] when the bacteria are grown under specific culture conditions, leading to a lower apparent plasmid-to-chromosome ratio even if each cell contains at least one plasmid. This study also indicates that the copy number can vary depending on the growth phase, highlighting that plasmid-to-chromosome ratios are influenced by both chromosomal multiplicity and the physiological state of the cells. Alternatively, population heterogeneity or asynchronous replication between the chromosome and plasmid could result in transient variations in plasmid copy number.

Crystal Digital PCR^TM^ is an effective technique for quantifying gene copy numbers and has the potential to be widely applied in various areas, including the quantification of transposable element copies, copy number of genes encoding rRNA, etc. Transposable elements, which replicate through a copy-paste mechanism, are often present in large numbers within genomes. This abundance makes their accurate quantification challenging but now Crystal Digital PCR^TM^ can address this issue. At least, as Crystal Digital PCR^TM^ allows for the quantification of gene copies relative to the number of chromosomes, this technique can be applied to measure horizontal gene transfer (HTG) and can be useful for studying, for example, the transfer of ICEs (Integrative and Conjugative Elements), plasmids via conjugation, and the dissemination of these genetic elements within a bacterial population.

## 5. Conclusions

In conclusion, the Crystal Digital PCR^TM^ method is a powerful and reliable approach for quantifying bacterial populations, even in complex settings such as mixed communities. Its high sensitivity and precision make it particularly well suited for synthetic microbial consortia, where the species composition is predefined and primers can be carefully designed to target specific sequences. This targeted approach allows for accurate quantification and monitoring of each member within the consortium, facilitating detailed studies of microbial interactions and dynamics. However, when applying dPCR to natural or environmental consortia, its utility becomes more limited. The inherent complexity and diversity of these communities, combined with incomplete or insufficient knowledge of the species present, pose significant challenges for primer design. Without comprehensive genomic information, there is an increased risk of cross-hybridization and non-specific amplification, which can lead to erroneous quantification and misinterpretation of species abundance. Moreover, as dPCR relies on DNA amplification, it cannot differentiate between DNA originating from live, metabolically active cells and DNA from dead or dormant bacteria. Consequently, dPCR may detect residual DNA from species that are no longer viable or actively participating in the community, thus reflecting historical presence rather than current activity. This limitation is particularly critical in environments where DNA persists for extended periods after cell death, potentially biasing assessments of community structure and functional dynamics.

## Figures and Tables

**Table 1:** Oligonucleotides used for DNA quantification.

*N. vulgaris*	Primer Name	Sequence
Genes		
*DVU_16S*	oLD_DVU_16S_Fwd_	TCTGGCTCAGATTGAACGCT
	oLD_DVU_16S_Rev_	GCAAGCAGAGGCCACCTTTC
*DVU_0169*	oLD_DVU_0169_Fwd_	AAGAAGTTCCCCCAGTTCGC
	oLD_DVU_0169_Rev_	TTGTCGAGATTGTAGCGGGG
*DVUA0034*	oLD_DVUA0034_Fwd_	GGGTTGGTCGAGAAGTGGTT
	oLD_DVUA0034_Rev_	AGTTGCAGGAGAAGTACGGC
***C. acetobutylicum***	**Primer Name**	**Sequence**
Genes		
*CA_16S*	oLD_CA_16S_Fwd_	CAGGATGACAGGTGGTGCAT
	oLD_CA_16S_Rev_	AGCCCTAGACATAAGGGGCA
*CA_C0825*	oLD_CA_C0825_Fwd_	AGAGACACCGGTGCAAAGAA
	oLD_CA_C0825_Rev_	CTTTGCGCTTCCCCAAGATG
*CA_P0035*	oLD_CA_P0035_Fwd_	AGTGCCGCATCCAAGAGTAA
	oLD_CA_P0035_Rev_	ATGCCTTCTTCACAGGGAGC

## Data Availability

The original contributions presented in this study are included in the article. Further inquiries can be directed to the corresponding authors.
